# *Chlamydia trachomatis* Whole-Proteome Microarray Analysis of The Netherlands Chlamydia Cohort Study

**DOI:** 10.3390/microorganisms7120703

**Published:** 2019-12-16

**Authors:** Katrin Hufnagel, Bernice Hoenderboom, Christoph Harmel, Juliane K. Rohland, Birgit H.B. van Benthem, Servaas A. Morré, Tim Waterboer

**Affiliations:** 1 Infections and Cancer Epidemiology, German Cancer Research Center (Deutsches Krebsforschungszentrum, DKFZ), 69120 Heidelberg, Germany; harmel@stud.uni-heidelberg.de (C.H.); rohland@stud.uni-heidelberg.de (J.K.R.); t.waterboer@dkfz.de (T.W.); 2 Epidemiology and Surveillance Unit, Centre for Infectious Disease Control, National Institute for Public Health and the Environment, 3721MA Bilthoven, The Netherlands; birgit.van.benthem@rivm.nl; 3 Laboratory of Immunogenetics, Department Medical Microbiology and Infection Control, location VU University Medical Center, Amsterdam University Medical Centre (UMC), De Boelelaan 1108, 1081 HZ Amsterdam, The Netherlands; samorretravel@yahoo.co.uk; 4 Institute for Public Health Genomics (IPHG), Department of Genetics and Cell Biology, Research School GROW (School for Oncology & Developmental Biology), Faculty of Health, Medicine & Life Sciences, University of Maastricht, 6229 ER Maastricht, The Netherlands

**Keywords:** *Chlamydia trachomatis*, whole-proteome microarrays, serology, antigen identification

## Abstract

*Chlamydia trachomatis* (Ct) whole-proteome microarrays were utilized to identify antibody patterns associated with infection; pelvic inflammatory disease (PID), tubal factor infertility, chronic pelvic pain (CPP) and ectopic pregnancy in a subsample of the Netherlands Chlamydia cohort study. Serum pools were analyzed on whole-proteome arrays. The 121 most reactive antigens identified during whole-proteome arrays were selected for further analysis with minimized microarrays that allowed for single sera analysis. From the 232 single sera; 145 (62.5%) serum samples were reactive for at least one antigen. To discriminate between positive and negative serum samples; we created a panel of in total 18 antigens which identified 96% of all microarray positive samples. Antigens CT_858; CT_813 and CT_142 were most reactive. Comparison of antibody reactivity’s among women with and without Ct related sequelae revealed that the reactivity of CT_813 and CT_142 was less common among women with PID compared to women without (29.0% versus 58.6%, *p* = 0.005 and 25.8% versus 50.6%, *p* = 0.017 respectively). CT_858 was less common among CPP cases compared to controls (33.3% versus 58.6; *p* = 0.028). Using a whole-proteome array to select antigens for minimized arrays allows for the identification of novel informative antigens as general infection markers or disease associated antigens

## 1. Introduction

*Chlamydia trachomatis* (Ct) is the most frequently reported bacterial sexually transmitted infectious agent, and caused 127 million incident infections worldwide in 2016 [1]. Mostly women are affected, with an estimated worldwide prevalence of 4% among 15–49 years olds [1]. In women, Ct infections can ascend to the upper genital tract and cause pelvic inflammatory disease (PID), chronic pelvic pain (CPP), tubal factor infertility (TFI) and ectopic pregnancy (EP) [2].

Ct infections can be detected by either detecting the pathogen directly in clinical specimens, or by measuring antibodies against Ct in patient serum [3]. Nucleic acid amplification tests (NAAT) have replaced cell culture-based methods as the gold standard in clinical Ct diagnostics [4,5]. Although NAATs are highly suited to detect acute Ct infections, they are not able to detect past infections and may miss persistent, low load or upper genital tract infections. We hypothesize that when persistent upper genital tract infections are established and Ct resides in the form of aberrant reticulate bodies (RBs) inside epithelial cells, Ct as well as its DNA or RNA may no longer be detectable in swabs or other clinical specimens [6]. However, these infections may still be detectable indirectly through detection of Ct antibodies in serum [6].

The Microimmunofluorescence (MIF) test was formerly considered the gold standard for serodiagnosis of Ct infections [7]. In this test, serum antibodies against chlamydial elementary bodies (EB) are measured. Due to poor standardization, subjective microscopic readings and labor-intensive procedures of this method, alternative assay formats based on enzyme-linked immunosorbent assays (ELISA) have been developed [8]. However, most of these assays are limited to few defined chlamydial antigens. Commercially available assays use the Ct major outer membrane protein (MOMP), the heat shock protein 60 (hsp60), protease-like activity factor (CPAF) or translocated actin-recruiting phosphoprotein (TARP) as antigens [9,10]. The plasmid encoded Ct protein pGP3 is used in several published assays to detect Ct antibodies [11,12,13,14]. Another method to detect Ct antibodies to a large but still limited number of Ct proteins is multiplex serology, a bead-based high-throughput method used to detect antibodies to multiple antigens simultaneously [15]. This method has already been used to detect serum antibodies against selected Ct antigens in large cohorts [16,17].

Analysis of antibodies to the entire Ct proteome, consisting of 895 proteins, however allows the de novo identification of additional immunogenic Ct proteins. We recently developed a method to generate bacterial whole-proteome microarrays using a combination of Multiple Spotting Technique and cell-free, on-chip protein expression [18]. Bacterial proteins expressed on microarrays display antigenic epitopes, thereby providing an efficient method for immunoprofiling of patients and allowing de novo identification of antibodies associated with general infection as well as disease-related serum antibodies. Through comparison of antibody reactivity patterns, we identified antigens as markers for either general Ct infection or cervical cancer and validated these antigens using a high-throughput suspension bead array called multiplex serology [18]. De novo-identified disease-specific antibody profiles might be useful in clinical diagnosis, and allow the epidemiological investigation of the role of Ct infections in the development of different Ct-associated diseases.

In the Netherlands Chlamydia Cohort Study (NECCST), women of reproductive age were followed over time to assess Ct disease progression [19]. Serum samples from a subsample of NECCST participants were analyzed with Ct whole-proteome microarrays for the following aims: to identify informative antigens to distinguish between Ct infected and non-infected participants, and to explore associations between novel Ct antigens and PID, CPP, ectopic pregnancy and TFI. 

## 2. Methods

### 2.1. Study Population

Samples were selected from participants from NECCST. In NECCST, women of reproductive age are followed for a minimum of ten years until 2022 to assess Ct disease progression. NECCST participants previously participated in the Chlamydia Screening Implementation study (CSI) between 2008 and 2011 in which they participated in annual Ct NAAT tests [20]. In 2015–2016, these women were re-invited for NECCST. Participants filled in questionnaires concerning Ct infections, sexual risk behavior, PID, CPP, pregnancies and fertility. In case participants reported to be infertile they were asked how this diagnosis was made, the cause of infertility and possible treatments. Furthermore, additional informed consent was asked to request their medical files. Following the questionnaire, participants sent in a capillary blood sample to test for CT IgG to determine unnoticed infections using a Ct ELISA (CT IgG ELISA plus; Medac, Wedel, Germany).

### 2.2. Sample Selection

For the current study, we selected samples from a total of 5700 NECCST participants based on the following criteria: First, participants had given consent for the serum samples to be tested in other scientific research concerning sexually transmitted infections. Second, when sufficient amounts of serum volume, i.e., >24 µL were available. Lastly, when participants fell in any of the following three categories:

*Group 1.* Ct positive (i.e., tested positive by NAAT during the CSI, reported to have had a Ct infection, and/or tested positive for Ct IgG), but without any of the complications and at least once pregnant for a minimum of 20 weeks.

*Group 2.* Ct negative (i.e., no positive NAAT result during the CSI, no self-reported Ct infections and no Ct IgG antibodies), but with any of the complications, either PID, CPP, ectopic pregnancy or TFI.

*Group 3.* Ct positive (i.e., tested positive by NAAT during the CSI, reported to have had a Ct infection, and/or tested positive for Ct IgG) and with any of the complications, i.e., either PID, CPP, ectopic pregnancy or TFI.

In total, 259 serum samples were selected of which 143 (55.2%) belonged to group 1, 80 (30.9%) to group 2 and 36 (13.9%) to group 3. Characteristics of the selected study participants are shown in Table 1.

### 2.3. Whole-Proteome Microarray

Whole-proteome microarrays representing the entire proteome of Ct serovar D with 895 proteins were generated as previously described by Hufnagel et al. 2018 [18]. In order to generate Ct whole-proteome arrays, two successive PCRs were performed for all open reading frames (ORFs) based on Ct genomic DNA. The products of the second PCR carrying all sequences necessary for transcription and translation (T7 Promoter, untranslated region, ribosome binding site, start codon, T7 Terminator), fusion peptide tags (N-terminal 6x-His and C-terminal V5 tags) were directly (without purification) used as the expression constructs for cell-free expression.

Protein microarrays expressing in situ the entire Ct proteome were generated using Multiple Spotting Technique [21]. During the first spotting step, the products of the second PCR were transferred onto Ni-NTA slides using a Nanoplotter 2 (GeSIM, Radeberg, Germany). Subsequently, the S30 T7 High-Yield Protein Expression Kit (Promega, Madison, WI, USA) was transferred directly on top of the expression construct spots. The slides were then incubated overnight in a humidified environment and proteins were expressed directly on the microarray slide and immobilized to the nickel surface.

Success of protein expression on the microarray was determined by incubation with fluorescence-conjugated antibodies directed against the N- and C-terminal fusion tags (Anti-6xHis, DyLight 650 (Abcam, Cambridge, UK) and anti-V5, Cy3 conjugate (Sigma-Aldrich, St. Louis, Missouri, USA)). The slides were scanned on a Power Scanner (Tecan, Männedorf, Switzerland) at 532 nm and 635 nm excitation wavelengths, respectively, and analyzed using the microarray acquisition and analysis software GenePix Pro 6.0 (Molecular Devices, Sunnyvale, CA, USA). Signal intensities were measured as median fluorescence intensity (MFI) signal of all pixels measured for one protein. In addition to the 895 CT proteins, we included the Epstein–Barr virus (EBV) viral capsid antigen p18 (VCA) as a positive control. An EBV seroprevalence of around 95% in adults ensures a positive signal during proteome immunoassays (PIAs) even for Ct seronegative samples in the vast majority of cases. As a negative control (n.c.), both successive PCRs were performed without DNA template and the product of the second PCR was spotted as template for on-chip protein expression in one row of 20 replicates at the bottom of each slide. A protein was considered to be expressed if its signal intensity generated by the labeled antibodies to either the 6xHis or the V5 tag was higher than the mean plus five standard deviations of all n.c. replicates.

PIAs were performed by incubating serum samples in pools of five. These pools of five sera of 6 µL each were generated based on long-term complications and if possible on different age categories of participants. In total, we had 53 pools either Ct positive with sequelae (separately for PID, CPP, ectopic pregnancy or TFI) or Ct negative with sequelae (Supplementary Figure S1). Because of the large sample, CT positive women without sequelae were pooled in different age categories i.e., < 30, 30–32, 33–35, and >35 years. Of 53 pools, 48 were included in the PIA because of batch size. Five pools of controls were randomly excluded. Binding of a serum antibody to an antigen on the microarray was detected by a secondary fluorescence-conjugated antibody (Alexa Fluor 647-conjugated goat anti-human IgA, IgG, IgM; Jackson Immuno Research, West grove, PA, USA). The microarrays were scanned at an excitation wavelength of 635 nm. The signal intensity obtained for a given antigen was considered proportional to the amount of primary antibody bound on the microarray.

Quality (QC) control of PIAs was performed by assessing the scanned images of each slide, and representing each PIA as a plot in which the acquired MFI data is visualized according to its position on the slide.

This allows for the recognition of technical artefacts on the individual slides, such as gradient effects of the background or MFI signals. Whole-proteome slides were excluded if they met one of the following criteria:

(i) gradient effects (e.g., loss of reactivity from top to bottom of a slide);
(ii) smearing of spots (due to e.g., insufficient washing);
(iii) disproportional high readouts for all negative controls (leading to the detection of false negative readouts of antigens).

As QC procedures were qualitative in nature, they were performed independently by two scientists (KH and CH).

### 2.4. Selection of Informative Antigens

The files generated with GenePix Pro 6.0 contained the MFIs values for each processed slide and were analyzed using the statistical programming language R (R Core Team, 2018).

For analysis of the PIAs, data were imported to R and seropositivity was determined by neighborhood averaging, i.e., by calculating a specific threshold for each spot position in order to address local variation of the signal intensity across the array (Equation (1)). The threshold criterion takes the relative distances of the spots into account, so that for the calculation of one spot’s threshold the MFI values of the 50 closest spots are considered, not distinguishing between negative controls and protein spots but excluding positive controls.

*Seropositive = MFI_spot_ > (MED(MFI_50 closest spots_) + 3MAD(MFI_50 closest spots_))* *MED = median, MAD = median absolute deviation* (1)

Different approaches to calculate a threshold were evaluated in order to determine the most robust approach. The calculation and comparison of three different threshold criteria are described in Supplementary Methods S1. After removing slides from the dataset based on the described quality control criteria, Ct proteins were selected for further spotting of microarrays that allow for the incubation of 8 single sera per slide. This new spotting layout limits the number of spots per block to 140 (121 Ct proteins, 4 positive and 15 negative controls). The 120 most reactive antigens identified during incubation of serum pools on whole-proteome microarrays were included in the minimized microarray. In addition, CT_798 was included since it was identified during a previous study using Ct whole-proteome microarrays and was already validated using multiplex serology [18]. This resulted in a total number of 121 antigens. Although antibodies that exceeded the threshold-fold only once might be of low prevalence or present only in low titers, they might nevertheless be promising markers for general infection or disease association. The 121 antigens were expressed on microarrays containing eight blocks separated by frames. Immunoassays as well as quality controls were performed as described for whole-proteome microarrays. Since the 15 negative controls were distributed over the whole slide a weighted negative control approach was used. For each spot, an individual threshold is calculated based on distance-weighted negative control signals. The threshold *t* is defined for each spot *j* as (Equation (2)):

(2) $$t_{j}={\bar{z}}_{j}+3\cdot \sqrt{{\hat{\sigma }}_{j}^{2}}$$

With ${\bar{z}}_{j}$ being the weighted mean of the MFI values of the negative controls $z_{i}$ and ${\hat{\sigma }}_{j}^{2}$ being the respective weighted variance. The weights $w_{i,j}$ are defined as the reciprocal euclidean distances of the negative controls $z_{i}$ to the spots *j* with x and y being the respective cartesian coordinates of the negative controls *i* and spots *j* on the array (Equations (3)–(5)):

(3) $$w_{i,j}={\sqrt{{\left(x_{j}-x_{i}\right)}^{2}+{\left(y_{j}-y_{i}\right)}^{2}}}^{-1}$$

(4) $${\bar{z}}_{j}=\frac{\sum_{i=1}^{n}w_{i,j}z_{i}}{\sum_{i=1}^{n}w_{i,j}}$$

(5) $${\hat{\sigma }}_{j}^{2}=\frac{\sum_{i=1}^{n}{\left(z_{i}-{\bar{z}}_{j}\right)}^{2}}{\sum_{i=1}^{n}w_{i,j}}$$

### 2.5. Statistical Analyses

Descriptive analyses were performed and characteristics of the study population were presented.

1. Reactivity of the antigens.

Percentage positive was described for serum samples positive for at least one antigen up until at least five positive antigens in the minimized array. Furthermore, a panel of the most distinctive antigens to capture all the serum samples positive for at least one antigen were determined. Last, the fifty most reactive antigens were identified based on the number of positive sera.

2. Exploration of Ct antigens related to sequelae.

The percentage positive, the median number of positive antigens and the most frequent found antigens (three highest percentages of at least 40%) were described for Ct positive without complications (group 1), Ct negative with complications (group 2), and Ct positive with complications (group 3). Proportions of positive serum samples against specific antigens were compared between PID-, CPP-, EP- and TFI-cases and controls (Ct positive without complications) using chi-squared tests.

Statistical analyses were performed using R studio and STATA (Version 14.2; StataCorp, College Station, TX, USA).

### 2.6. Ethics Approval

This study was approved by the Medical Ethical VU Medical Center, Amsterdam, the Netherlands (NL 51553.094.14/2015.903(A2019.336)). Date of approval Committee 10/13/2015. All participants provided informed consent.

## 3. Results

### 3.1. Antigen Selection

**Figure 1:**
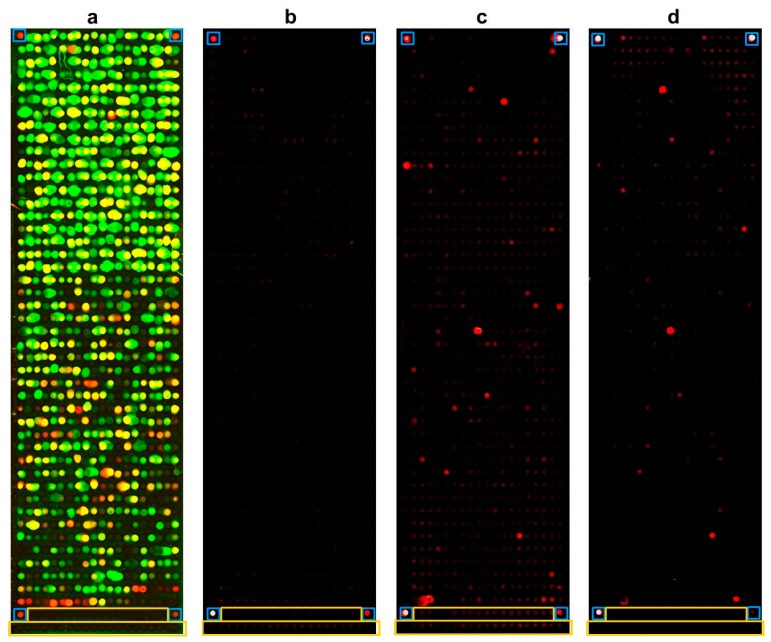
Proteome immunoassays (PIA) using pools of five sera. In total, 895 Ct proteins were spotted on one array. Negative and positive controls are highlighted with yellow and blue boxes, respectively. (**a**): Expression control using fluorescence-conjugated antibodies against the terminal tags of the Ct proteins (green signal: anti-V5 antibody; red signal: anti-His antibody; yellow signal: overlay of both signals). (**b**): No signal was obtained with a pool of five sera from Ct-uninfected women for any of the Ct antigens but showed reactivities with all four positive controls. (**c**,**d**): Results of two PIAs with pools of five sera from women infected with Ct.

**Figure 2:**
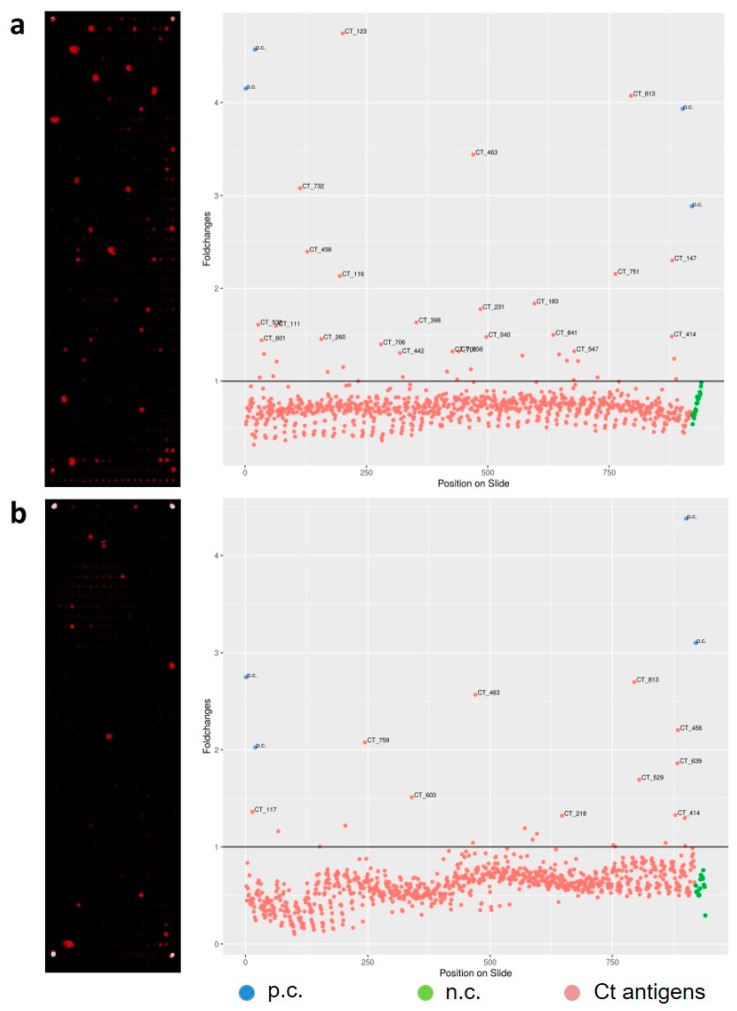
Result of two PIAs using pools of five sera from Ct-infected patients with (**a**) EP and (**b**) PID. Pictures on the left show scanned images of microarray slides after performing PIAs. Red and white spots indicate reactivities of the incubated serum pool with the antigen expressed on this position of the slide. The graphs on the right side illustrate signal intensity by Foldchanges of each antigen. Antigens are plotted onto the x-axis based on their position on the microarray slide. The threshold is illustrated by a straight black line in the graph. Individual Ct proteins with elevated reactivity are labeled with individual Ct ORF numbers. The Epstein–Barr virus viral capsid antigen was spotted as a positive control (p.c., blue) in all four corners. Negative controls (n.c., green) were spotted in the last row of each slide. An antigen was considered to be immunogenic if its signal was higher than the defined threshold (indicated by the black line y = 1).

We were able to successfully express more than 96% of all 895 Ct proteins. The result of the expression staining using labelled antibodies against the terminal fusion tags of the 895 Ct proteins is illustrated in 2a. In total, PIAs were performed for 48 serum pools of the NECCST study. Exemplary results for three PIAs are illustrated in Figure 1b–d. Of these 48 slides, six were removed after quality control. Two slides were removed due to vertical gradients based on incomplete protein expression, and two other slides showed horizontal gradients probably due to insufficient washing; two additional slides did not pass quality control because of extensive smearing between spots. Seventeen of the analyzed pools only comprised sera from women who were considered Ct negative based on previous tests or questionnaire. The serum samples were nevertheless included, since they were all collected from women with Ct associated complications. Only five of these pools were identified as truly negative on the Ct whole-proteome microarray. Based on the results of the 42 remaining whole-proteome arrays (Figure 2), we selected the 121 most reactive and thus potentially informative antigens. No differences were seen between the different pools. The 121 most reactive antigens represent 13.5% of the complete Ct proteome. Fourteen of the 121 antigens showed N-terminal but no C-terminal expression signals on whole-proteome arrays. Although no C-terminal expression signal was detected, these 14 antigens were nevertheless recognized by serum antibodies of Ct-infected individuals at least once. A list of the 120 most reactive Ct antigens and their corresponding threshold-fold values can be found in Supplementary Table S1. CT_798 is not included in the table since it was not identified as a reactive antigen in this study but was added due to previous findings.

### 3.2. Single Sera Outcomes

**Figure 3:**
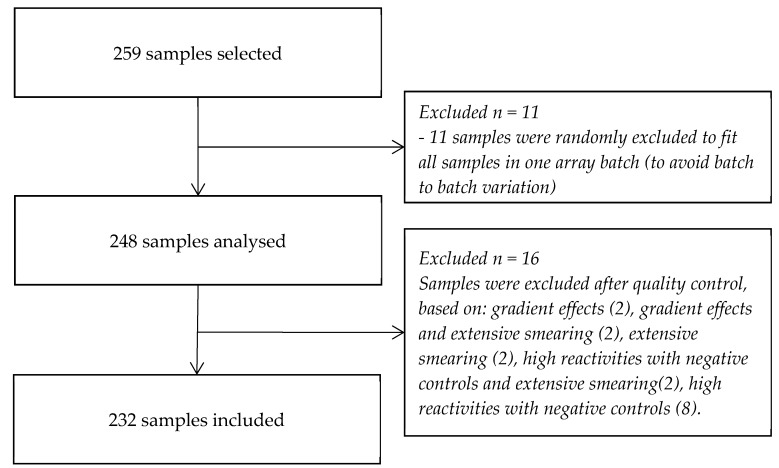
Flowchart of the included samples.

**Figure 4:**
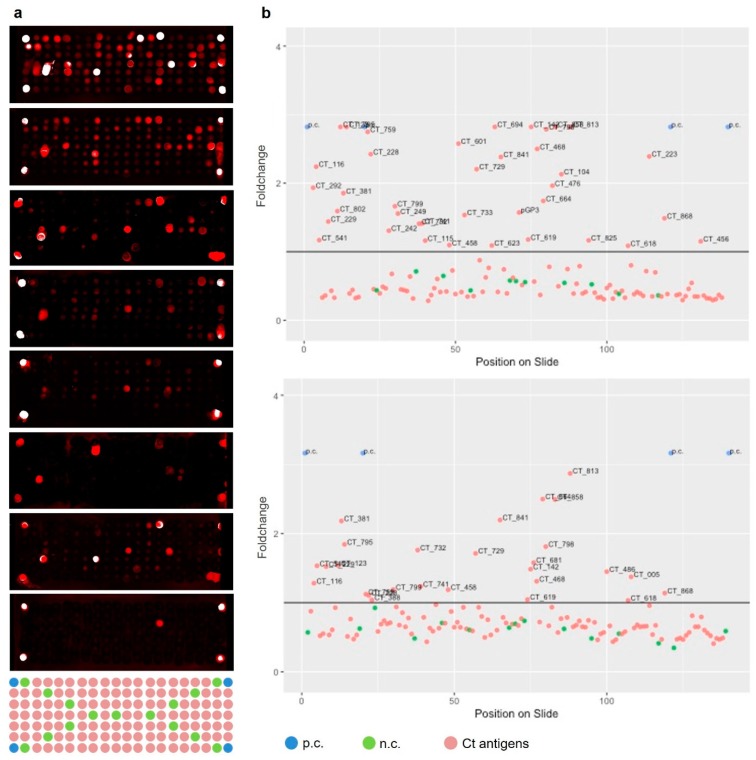
Results obtained after performing immunoassays with single serum samples on minimized arrays containing 121 identified Ct antigens. (**a**): scanned images of immunoassays with 8 single sera from Ct-infected women and layout of each of the eight blocks. Red and white spots indicate reactivities of the incubated serum sample with the antigen expressed on this position of the slide. (**b**): data analysis for the two topmost blocks in panel a. The graphs illustrate signal intensity by Foldchanges of each antigen. Antigens are plotted onto the x-axis based on their position on the microarray slide. The threshold is illustrated by a straight black line in the graph. Individual Ct proteins with elevated reactivity are labeled with individual Ct ORF numbers. The Epstein–Barr Virus Viral Capsid Antigen was spotted as a positive control (p.c., blue) in all four corners. Negative controls (n.c., green) were spotted throughout each block. An antigen was considered to be immunogenic if its signal was higher than the defined threshold (indicated by the black line y = 1).

Of 259 selected serum samples, 248 single samples were tested and following quality control, 16 were excluded, (Figure 3). No significant differences in characteristics were found between the initial 259 samples and the in total 232 single included sera. Typical results and corresponding data analysis are shown in Figure 4.

1. Reactivity of the antigens

**Figure 5:**
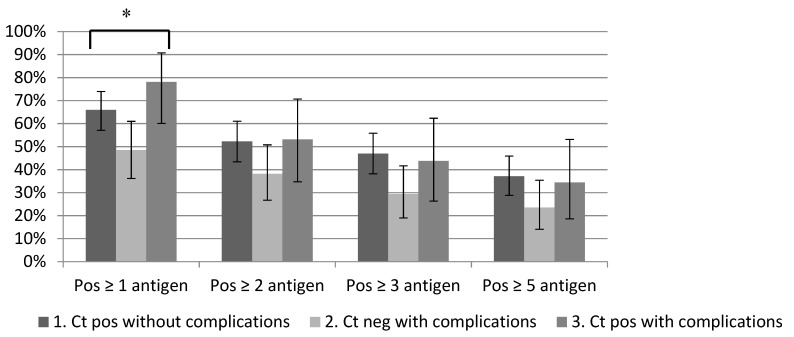
Percentage positive by minimized array between Ct positive without complications, Ct negative with complications and Ct positive with complications. * = a significant difference (*p* < 0.05) in percentage positive.

From the 232 single sera, 145 (62.5%) serum samples were reactive for at least one antigen and 76 (32.8%) serum samples for at least five antigens (Figure 5). The median number of reactive antigens was five (IQR 2-11) (Table 2). The percentage of reactive sera was highest among group 3: Ct positive women with complications group 3 (78.1%), while among group 1 (Ct positive controls) 65.9% of all tested sera showed reactivity with at least one antigen. Lowest reactivities were observed among group 2 (Ct negative women with complications) which was 48.5%, *p* = 0.008. Using a stricter definition of seropositive (i.e., at least positive against two, three or five antigens) showed similar patterns though with lower percentage of reactive sera and no significant difference between the three groups. Among serum samples from women that were previously tested as Ct-NAAT positive, 81.3% showed reactivity with antigens on the microarray. This was 78.2% among MOMP ELISA positives and 55.7% among self-reported positives only, *p* = 0.001. Of previously Ct-NAAT negatives, 51.5% were negative in the minimized array.

**Figure 6:**
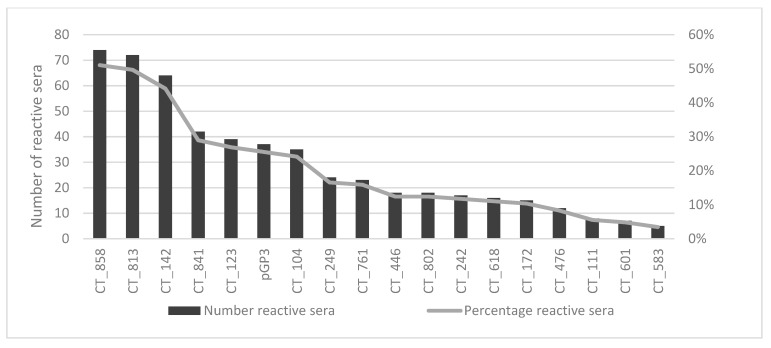
Panel of 18 antigens to identify 96% of all positive serum samples in the cohort.

To discriminate between serum samples positive for at least one antigen and serum samples negative for all antigens, we created a panel of in total 18 antigens which can identify 96% of all serum samples positive for at least one antigen, Figure 6.

The fifty most reactive antigens are shown in Table 3. CT_858 (CPAF) and CT_813 were most reactive and positive in 51% and 49% among serum samples positive for at least 1 antigen, respectively. Antigen CT_142 was reactive in 44% of the positive samples. Besides CPAF other known immunogenic antigens such as pGP3, CT_681 (MOMP), CT_456 (TARP), and CT_110 (Hsp60) were also among the fifty most reactive antigens. 

2. Exploration of Ct Antigens Related to Sequelae

Of eleven TFI cases, nine (81.2%) were positive for antibodies against at least one antigen, most frequently CT_142 and CT_858 (Table 2). Thirty out of forty-six PID cases were positive against at least one antigen (67.4%), which was most often CT_858. Among CPP and EP cases, 54.5% and only 27.3% were positive for at least one antigen, respectively.

Table 4 shows the presence of antibodies against antigens that were significantly different between cases (i.e., PID or CPP positive) and controls (Ct positive without any complication). The presence of antibodies against CT_813 and CT_142 was less common among women with PID compared to women without PID. In CPP positive women, the presence of antibodies against CT_858 was less common compared to women without CPP. No significant differences in the presence of specific antibodies were found between TFI or EP cases compared to controls.

## 4. Discussion

Using Ct whole-proteome microarrays and minimized arrays, we aimed to identify informative antigens to distinguish between Ct infected and non-infected women and to identify antigens associated with Ct-associated disease outcomes. To this end, we analyzed 232 samples from the NECCST study, a cohort study which follows women of reproductive age to assess Ct disease progression. We found 145 (63%) serum samples to be positive for at least one antigen in the minimized array. The antigens CT_858, CT_813 and CT_142 were most reactive in our sample of women. Among PID positive women, fewer serum samples were positive for the antigens CT_142 and CT_813 compared to women without PID. In CPP positive women, CT_858 was less common compared to controls.

In the Ct whole-proteome arrays, 860 out of all 895 Ct proteins were successfully expressed. Of all expressed Ct proteins, 121 antigens were selected for the minimized array based on analysis of pools of sera. Using minimized arrays that included the 121 selected antigens, 232 single serum samples were tested. Overall, 63% of sera were positive for at least one of these 121 antigens. In sera from women who previously tested positive for Ct by NAAT, 81% were positive for at least one antigen. Considering that women were Ct NAAT positive between 2008 and 2011, that antibodies can wane over time, and given not every infected woman seroconverts [11,22], this is a high agreement. This agreement is higher than other published serological assays which utilize only one or few Ct antigens for serodiagnosis of Ct infections. In a study in which five Ct antibody assays with different target antigens were compared, seropositivity was only between 38% and 68% after a median of three years and a minimum of six months after infection [23]. On the other hand agreement with Ct NAAT negatives was only 51.5%, because 48.5% of previously Ct-NAAT negatives were reactive for at least one antigen in the minimized array. This is difficult to interpret. Although NAAT tests are very sensitive and specific, the test can only measure current infections and does not rule out past infections [3]. In addition, samples from these women were tested with the Medac MOMP ELISA, but this assay only detects about 40% of past infections [23,24,25]. Furthermore, these Ct-NAAT negative tested women, were all women with CPP, PID, ectopic pregnancy or TFI, complications which could relate to Ct infections and thereby possible exposed to past Ct infection. Nevertheless, it is unlikely that all these 48.5% microarray positives are true positives. For further research this array has to be tested in a population with true or reliable Ct negatives, for example in blood samples from infants or children or nuns.

In our cohort, CT_858 and CT_813 were identified as the most reactive antigens and showed reactivities with 51% and 49% of all Ct seropositive sera, respectively. While CT_858 alone would react only with 51% of all Ct seropositive samples, a combination of CT_813 and CT_858 would result in a 65% detection rate. Furthermore, a panel of CT_858, CT_813, CT_142 and CT_104 would be able to identify 78% of all seropositive samples. This means, we observe a higher coverage with more antigens and the coverage rate cumulatively increases with number of antigens due to different reactivity patterns of individual samples. Therefore, we suggest a panel of 18 antigens to identify 96% of all Ct seropositive sera. Each of the six serum samples not reacting with any of the 18 antigens shown in Figure 6 showed only extremely weak reactivities with a single additional antigen. These reactivities might be unspecific signals. However, it cannot be excluded that these sera are true Ct seropositive samples and we miss them with our panel of 18 antigens. An ideal platform for such a serological assay would be high-throughput bead-based multiplex serology. For the bacterium *Helicobacter pylori* a panel of 17 antigens is already widely used to detect differential antibody responses [26].

CT_858 is a serine protease known as chlamydial proteasome/protease-like activity factor (CPAF) which is secreted from the chlamydial intracellular inclusion into the host cell cytoplasm [27]. The role of CPAF as a virulence factor that degrades host proteins is currently widely discussed [28,29]. As a secreted virulence factor that is expressed during Ct infection, CPAF is likely a target for host antibodies and was therefore already used in published serological assays [30,31]. The hypothetical protein CT_813 was identified as an antigen to determine general Ct infections and was described by Chen et al. to be located within the Ct inclusion membrane. They were able to detect the protein 12 h after chlamydial infection. In addition, it was shown that CT_813 is present in the inclusion membrane during the entire growth cycle and an interaction of this protein with host cells was described [32]. CT_813 is expressed in many Ct serovars but not in other chlamydial species, which makes it a promising candidate to specifically detect Ct infections without any cross-reactivity to other species. Another promising antigen identified during this study to detect general Ct infections is the hypothetical protein CT_142. So far, and to the best of our knowledge, nothing is published about its function and role in the Ct infection cycle. Previously, CT_813 and CT_142 were validated as general infection markers in a large seroepidemiological study using multiplex serology [18]. Among women positive for at least one antigen in our study, antibodies against antigens CT_813 and CT_142 were less common among PID cases compared to women without PID. This might indicate an effective immune response that may render a protective effect against PID. Among CPP cases, CT_858 was less common compared to women without CPP. More research is needed to gain insight in the functions and roles of CT_813, CT_142 and CT_858 in Ct-associated disease. Disease specific antigens for EP or TFI were not found.

Besides the identification of novel immunogenic antigens we were able to confirm known immunogenic antigens as infection markers, like the major outer membrane protein (CT_681), the plasmid encoded protein pGP3, and Hsp60 (CT_110) all of which are already used in both commercial and non-commercial serological assays to detect Ct serum antibodies [11] The most reactive antigens (CT_858, CT_813, CT_142, CT_841, CT_795, CT_123, pGP3) which reacted with more than 25% of all tested sera, were also identified as highly immunogenic antigens when analyzing serum samples from the Mongolian population based cross-sectional HPV prevalence study on Ct whole-proteome microarrays [18]. The identification of the same set of most reactive antigens in two independent studies indicates that these antigens are promising markers to detect general Ct infections.

In total 63% of all tested sera reacted at least with one Ct antigen on the microarrays. The humoral immune system of these Ct infected women recognizes and responds to different Ct antigens by eliciting a variety of antibodies. The remaining 38% (*n* = 87) of the tested serum samples did not result in significant signals, although 52 (60%) of these were from women previously tested positive for Ct (either self-report or study NAAT) or ELISA assay. The immune system of these individuals did not produce antibodies against Ct antigens included in our assay. However, these women showed a specific reactivity with the positive control (EBV VCA) as illustrated in Figure 4. We observed an EBV seroprevalence of >90% in minimized arrays both for Ct-infected as well as Ct-uninfected sera, which corresponds well to the expected seroprevalence in adults of around 95%.

### Limitations

The major outer membrane protein MOMP (CT_681) reacted only with 20 analyzed serum samples in this study. Since this antigen is used in many commercially available assays, we expected to detect reactivities to this particular antigen more often. However, these assays often utilize linear peptides that might, in this case, be a better target for serum antibodies. If the full length MOMP on the microarray is incorrectly folded, the epitope might not be accessible to the serum antibodies. Incorrect protein folding or aggregation might result in false negative signals and therefore some immunogenic antigens might not be detectable on the microarray. However, since we were able to detect high signals for antigens such as CT_858, CT_813 and CT_142 this may be a limitation for some but not for all antigens.

Second, we had a small sample size to identify disease specific antigens, especially for EP and TFI which consisted of only three and nine seropositive serum samples, respectively. This limitation makes it impossible to come to definitive conclusions about associations between specific antigens and these Ct outcomes.

The third limitation is the possibility of antibody cross-reactivity. In order to investigate cross-reactivities of the identified antigens with antibodies to antigens of the closely related organism *Chlamydophila pneumoniae* (Cp), homologies to Cp proteins were analyzed using the NCBI protein blast tool (https://blast.ncbi.nlm.nih.gov/Blast.cgi?PAGE=Proteins). The hypothetical protein CT_142 showed an identity of 35% to a hypothetical protein of Cp while CT_858 showed 48% sequence identity to CPAF of Cp. Therefore, cross-reactivities cannot be excluded for these antigens, although substantial cross-reactivity usually requires a higher degree of amino acid relatedness. However, CT_813 showed no homologies to any protein of Cp. Therefore, cross-reactivity for CT_813 with antibodies against Cp antigens is unlikely. Nevertheless, these proteins should be validated in a cohort with serum samples that are truly seronegative for Ct and positive for any of the other chlamydial species to test possible cross-reactivity. In addition, highly immunogenic proteins of closely related species, like the major outer membrane protein of Cp, should be included in whole-proteome and minimized arrays to test for possible cross-reactivities.

## 5. Conclusions

Using a whole-proteome array to select antigens for minimized arrays allows for the identification of novel and potentially promising antigens to distinguish between Ct negative and Ct positive women. In our minimized Ct array containing 121 antigens, seropositivity was 81% in women previously tested Ct NAAT positive. A panel of 18 antigens of these 121 antigens can identify 96% of all microarray seropositive samples in this cohort. Antigens CT_858, CT_813 and CT_142 were most reactive and might be of value for future Ct antibody assays.

## Figures and Tables

**Table 1:** Characteristics of the study population.

	Overall N = 259	Ct Positive Without Complications N = 143 (55.2)	Ct Negative with Complications N = 80 (30.9)	Ct Positive with Complications N = 36 (13.9)
	n (%)	n (%)	n (%)	n (%)
Age (years)				
<30	77 (29.7)	41 (28.7)	27 (33.8)	9 (25.0)
30–32	69 (26.6)	31 (21.7)	22 (27.5)	16 (44.4)
33–35	75 (29.0)	45 (31.5)	23 (28.8)	7 (19.4)
≥36	38 (14.7)	26 (18.2)	8 (10.0)	4 (11.1)
CT history				
Negative	80 (30.9)	0 (0.0)	80 (100.0)	0 (0.0)
Positive by at least NAAT ^$^	32 (12.4)	25 (17.5)	0 (0.0)	7 (19.4)
Positive by at least MOMP ELISA ^o^	71 (27.4)	56 (39.2)	0 (0.0)	15 (41.7)
Positive by only self-reported infections	76 (29.3)	62 (43.4)	0 (0.0)	14 (38.9)
Ct complications *				
None	143 (56.9)	143 (100.0)	0 (0.0)	0 (0.0)
PID	52 (20.1)	0 (0.0)	31 (38.8)	21 (58.3)
CPP	54 (20.9)	0 (0.0)	39 (48.8)	15 (41.7)
EP	11 (5.5)	0 (0.0)	9 (11.3)	2 (5.6)
TFI	13 (5.0)	0 (0.0)	8 (10.0)	5 (13.9)

^$^ All women with a positive NAAT result were included. ^o^ All women with a positive MOMP ELISA test, excluding women with a positive NAAT, were included. * Numbers do not add up to 100%, i.e., women could have multiple sequelae. IQR = interquartile range. PID = pelvic inflammatory disease, CPP = chronic pelvic pain, EP = ectopic pregnancy, TFI = tubal factor infertility, MOMP = major outer membrane protein, ELISA = enzyme-linked immunosorbent assays.

**Table 2:** Percentage positive serum samples by different subgroups.

	Positive Serum Samples N (%, 95%CI)	No. of Positive Antigens Median (IQR)	Top Three Antigens with Highest Seroprevalence (≥40%) %
**Controls—Ct positive**	87 (65.9, 57.2–73.9)	5 (2–15)	CT_858 (58.6), CT_813 (58.6), CT_142 (50.6),
**Cases—Ct negative**	33 (48.5, 36.2–61.0)	4 (2–7)	-
PID positive	28 (57.1, 37.2–75.5)	3.5 (2–6)	-
CPP positive	13 (43.3, 25.5–62.6)	5 (1–6)	-
EP positive	2 (22.2, 2.8–60.0)	10.5 (7–14)	NA
TFI positive	4 (66.7, 22.3–95.7)	7 (2–14)	CT_123 (50.0), CT_142 (50.0), CT_664 (50.0), CT_858 (50.0), CT_104 (50.0), CT_813 (50.0)
**Cases—Ct positive**	25 (78.1, 60.0–90.7)	3 (1–9)	CT_142 (56.0), CT_858 (52.0), CT_813 (44.0)
PID positive	15 (83.3, 58.6–96.4)	2 (1–9)	CT_858 (53.3), CT_142 (40.0), CT_813 (40.0),
CPP positive	11 (78.6, 49.2–95.3)	6 (1–11)	CT_142 (54.5), CT_841 (45.5), CT_858 (45.5), CT_813 (45.5)
EP positive	1 (50.0, 1.25–98.7)	1	NA
TFI positive	5 (100.0)	6 (2–9)	CT_142 (100.0) *, CT_858 (60.0) *

Controls—Ct positive = tested positive for Ct by NAAT, self-reported positive test or tested positive in Medac Momp assay but without any complication. Cases—Ct negatives = never tested positive for Ct by NAAT, no self-reported infections and negative for Ct antibodies by Medac Momp assay but with any of the complications (i.e., PID, CPP, EP or TFI). Ct positives = tested positive for Ct by NAAT, self-reported positive test or tested positive in Medac Momp assay but with any of the complications. PID = pelvic inflammatory disease, CPP = chronic pelvic pain, EP = ectopic pregnancy, and TFI = tubal factor infertility. IQR = interquartile range. * = only the first two highest percentages were described. NA = not applicable (sample size too small).

**Table 3:** The fifty most reactive antigens. CPAF = protease-like activity factor.

	Antigen	Name	No Reactive Sera	%		Antigen	Name	No Reactive Sera	%
1	CT_858	**protease-like activity factor CPAF**	74	51.0%	26	CT_228	hypothetical protein	17	11.7%
2	CT_813	hypothetical protein	72	49.7%	27	CT_664	hypothetical protein	16	11.0%
3	CT_142	hypothetical protein	64	44.1%	28	CT_798	glycogen synthase	16	11.0%
4	CT_841	ATP-dependent zinc metalloprotease FtsH	42	29.0%	29	CT_618	hypothetical protein	16	11.0%
5	CT_795	hypothetical protein	41	28.3%	30	CT_172	hypothetical protein	15	10.3%
6	CT_123	acetyl-CoA carboxylase biotin carboxyl carrier protein	39	26.9%	31	CT_687	cysteine desulfurase	14	9.7%
7	pGP3	**virulence plasmid protein**	37	25.5%	32	CT_456	**translocated actin-recruting phosphoprotein TARP**	14	9.7%
8	CT_104	enoyl-(acyl carrier protein) reductase	35	24.1%	33	CT_579	hypothetical protein	13	9.0%
9	CT_381	arginine ABC transporter substrate-binding protein ArtJ	35	24.1%	34	CT_117	inclusion membrane protein F	13	9.0%
10	CT_005	hypothetical protein	27	18.6%	35	CT_724	hypothetical protein	13	9.0%
11	CT_468	2-component regulatory system-ATPase	26	17.9%	36	CT_143	hypothetical protein	12	8.3%
12	CT_694	hypothetical protein	25	17.2%	37	CT_476	hypothetical protein	12	8.3%
13	CT_249	hypothetical protein	24	16.6%	38	CT_336	phosphoenolpyruvate-protein phosphotransferase	12	8.3%
14	CT_729	serine-tRNA ligase	24	16.6%	39	CT_110	**HSP60/chepronin GroEL**	11	7.6%
15	CT_761	peptidoglycan transferase	23	15.9%	40	CT_732	7-dimethyl-8-ribityllumazine synthase	11	7.6%
16	CT_223	inclusion membrane protein	21	14.5%	41	CT_332	pyruvate kinase	11	7.6%
17	CT_458	acetyltransferase	21	14.5%	42	CT_442	cysteine-rich protein	11	7.6%
18	CT_681	**major outer membrane protein**	20	13.8%	43	CT_529	hypothetical protein	10	6.9%
19	CT_802	S18 ribosomal protein	18	12.4%	44	CT_398	hypothetical protein	10	6.9%
20	CT_541	peptidyl-prolyl cis-trans isomerase	18	12.4%	45	CT_118	inclusion membrane protein G	10	6.9%
21	CT_229	hypothetical protein	18	12.4%	46	CT_868	deubiquitinase and deneddylase Dub1	9	6.2%
22	CT_446	hypothetical protein	18	12.4%	47	CT_799	50S ribosomal protein	9	6.2%
23	CT_116	inclusion membrane protein E	17	11.7%	48	CT_388	hypothetical protein	9	6.2%
24	CT_242	OmpH-like outer membrane protein	17	11.7%	49	CT_578	hypothetical protein	9	6.2%
25	CT_759	muramidase	17	11.7%	50	CT_226	hypothetical protein	9	6.2%

Antigen names presented in bold are antigens previously used in chlamydia immunoassays.

**Table 4:** The presence of specific antigens in controls and cases with pelvic inflammatory disease and chronic pelvic pain.

		Control *n* (%)	Case *n* (%)	*p*-value			Control *n* (%)	Case *n* (%)	*p*-value
**PID**					**CPP**				
	Antigen	PID negative	PID positive			Antigen	CPP negative	CPP positive	
	CT_813	51 (58.6)	9 (29.0)	0.005		CT_858	51 (58.6)	8 (33.3)	0.028
	CT_142	44 (50.6)	8 (25.8)	0.017					

PID = pelvic inflammatory disease, CPP = chronic pelvic pain. Control = Ct positive without any complication. Case = is either Ct positive of negative (in NECCST) but with PID or CPP.

## Supplementary Materials

The following are available online at https://www.mdpi.com/2076-2607/7/12/703/s1, Figure S1: Composition of serum pools, Methods S1: Calculation and comparison of three different threshold criteria, Table S1: The 120 most reactive antigens.
