# Supplementary material for: Chlamydia trachomatis Whole-Proteome Microarray Analysis of The Netherlands Chlamydia Cohort Study
Source: Microorganisms. 2019 Dec 16;7(12):703. doi: 10.3390/microorganisms7120703 (PMC6956083; doi:10.3390/microorganisms7120703)
Supplement: Supplementary file 1 [file microorganisms-07-00703-s001.pdf]

Supplementary material

- Page 2 - Supplementary Figure 1: Composition of serum pools
- Page 3 - Supplementary Methods 1: Calculation and comparison of three different threshold criteria
- Page 5 - Supplementary Table 1: The 120 highest reactive antigens selected from the whole proteome array

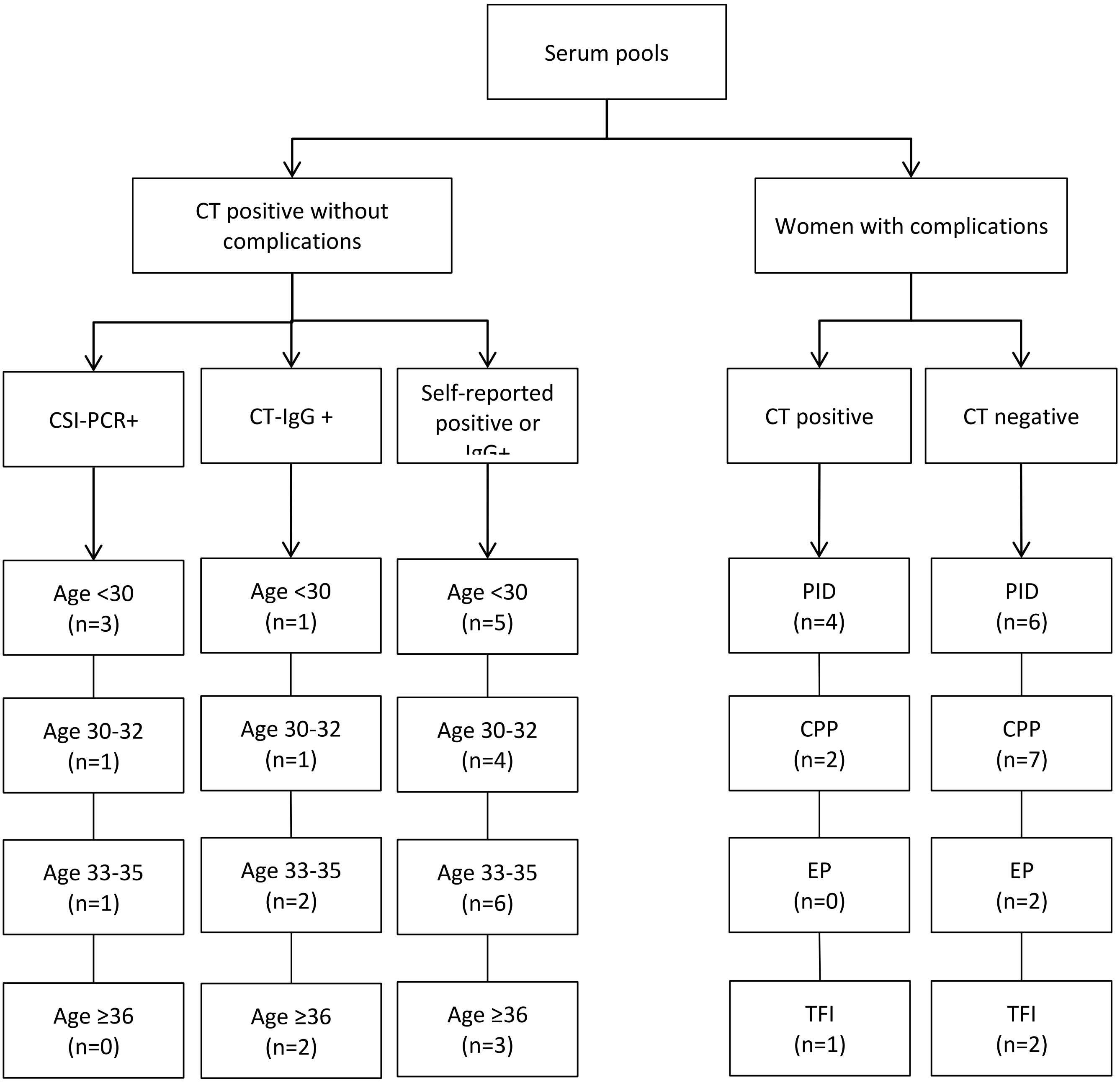

Figure 1 Composition of serum pools

Calculation and comparison of three different threshold criteria

For analysis of the proteome immunoassays, the acquired .gpr-files of all performed immunoassays were imported to R and different threshold criteria were calculated which are the following: the former used global threshold (Hufnagel et al., 2018) which considers a signal as significant if a MFI of a spot meets the following criterion:

$$(1)\quad MFI_{spot} > MFIs_{n.c.} + 5\ \sigma MFIs_{n.c.}$$

A second more robust global threshold criterion which utilizes the median (MED) and median absolute deviation (MAD):

$$(2)\quad MFI_{spot} > MED(MFI_{n.c.}) + 5\ MAD(MFI_{n.c.})$$

And a third approach in which seropositivity was determined by neighborhood averaging, i.e. by calculating a specific threshold for each spot position in order to address local variation of the signal intensity across the array. The threshold criterion takes the relative distances of the spots into account, so that for the calculation of one spot's threshold the MFI values of the 50 closest spots are considered, not distinguishing between negative controls and protein spots but excluding positive controls.

$$(3)\quad MFI_{spot} > MED(MFI_{50\ closest\ spots}) + 3\ MAD(MFI_{50\ closest\ spots})$$

An antigen is selected if it shows a given threshold-fold on any of the analyzed slides. The antigens were sorted by their maximal observed threshold-fold value and the top 120 antigens of that list are chosen for further analysis. Thereby, the applied threshold-fold criterion for seropositivity is set by the technical restrictions of the single sera analysis. Concerning this selection procedure, the global application of a threshold which is calculated from negative controls located on just one end of the slide might result in a biased selection. Therefore, antigens which are located in an area which possess high intensity signals will be

overrepresented in the above described selection list. In order to adjust for this issue individual thresholds were calculated for each spot by neighborhood averaging.

For each spot an individual threshold was calculated according to equation (3), whereas positive controls were excluded from the calculations. The effect of the neighborhood averaging method on threshold-fold data is shown in Supplementary Figure 1.

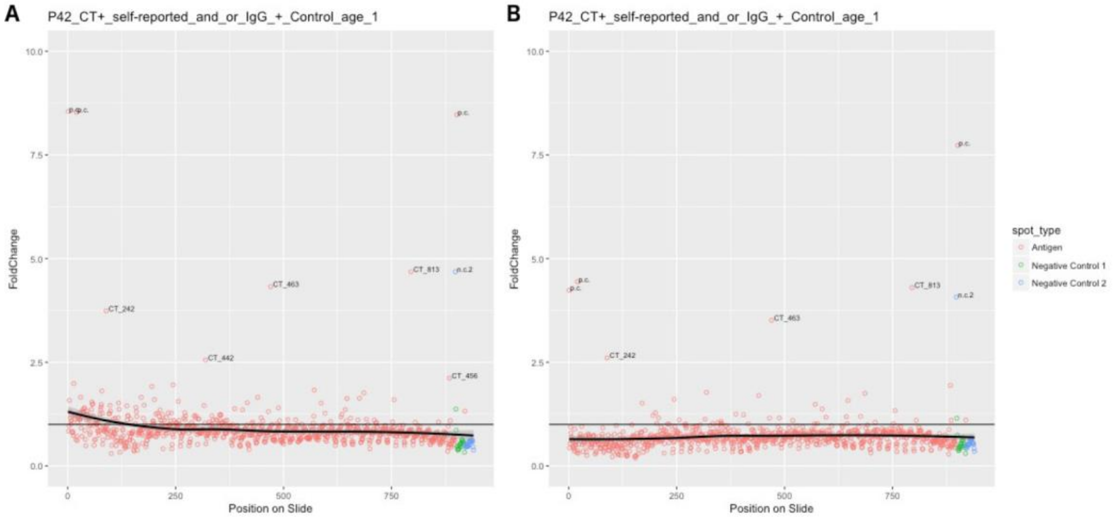

Supplementary Figure 1: Effects of neighborhood averaging on local signal in-varieties. All MFI-values are foldchange transformed after equation 2 (A) utilizing the median and mad of the negative controls and equation 3 which describes the neighborhood averaging (B) and plotted against the position on the slide (row-wise). Reactive antigens were labeled when they exceeded the fold-change of 1.7 which was chosen for illustration concerns of the plot.

In Supplementary Figure 1 A, the threshold-fold data calculated by the traditional method shows a gradient from the top to the bottom of the slide. After applying neighborhood averaging to the data (Supplementary Figure 1 B), the horizontal gradient disappeared. Selection lists were created using the global threshold and the spot individual neighborhood averaging method (see Supplementary Table 1). Both antigen lists were sorted by the maximal observed threshold-fold. The overlap between both lists was calculated and hits 51.67% at a list length of 120. The comparison of the selection list gained by applying a global threshold and by using the neighborhood method revealed that antigens with high reactivities are shared by both lists. The major differences lie within the lower ends of the lists which comprise reactivities that are slightly above threshold. Based on the knowledge of the presence of local

signal in-varieties, the list generated from the neighborhood averaging was chosen to be used when selecting the 120 antigens based on serum pool incubations on whole-proteome microarrays.

Supplementary Table 1: The 120 most reactive antigens

	Global Threshold				Neighborhood Averaging			
Rank	Antigen	max	mean	sd	ID	max	mean	sd
1	CT_813	7.282	1.401	1.469	CT_813	11.096	2.114	2.091
2	CT_463	6.362	1.053	1.202	CT_463	8.670	1.723	1.761
3	CT_242	4.830	1.004	0.943	CT_242	8.488	1.371	1.482
4	CT_123	3.889	0.626	0.902	CT_123	7.437	1.001	1.670
5	CT_456	3.253	0.735	0.671	CT_336	6.547	0.953	0.958
6	CT_183	2.850	0.647	0.444	CT_326	6.110	1.008	0.889
7	CT_442	2.552	0.691	0.498	CT_351	5.851	0.936	0.828
8	CT_868	2.419	0.664	0.480	CT_082	5.591	0.981	0.830
9	pGP3	2.377	0.486	0.452	CT_856	4.979	0.885	0.681
10	CT_732	2.369	0.648	0.509	CT_456	4.691	1.144	1.015
11	CT_858	2.363	0.662	0.464	pGP3	4.375	0.726	0.672
12	CT_759	2.181	0.684	0.474	CT_732	4.272	0.873	0.742
13	CT_618	2.157	0.603	0.369	CT_057	4.008	0.828	0.579
14	CT_555	2.071	0.498	0.316	CT_744	3.850	0.844	0.665
15	CT_040	2.039	0.485	0.312	CT_414	3.826	0.925	0.628
16	CT_746	2.003	0.492	0.371	CT_778	3.745	0.753	0.534
17	CT_117	1.987	0.467	0.366	CT_027	3.730	1.111	0.611
18	CT_467	1.975	0.655	0.457	CT_555	3.673	0.838	0.656
19	CT_027	1.927	0.675	0.417	CT_183	3.668	1.053	0.677
20	CT_822	1.920	0.464	0.336	CT_166	3.540	0.850	0.528
21	CT_116	1.914	0.618	0.349	CT_147	3.447	0.661	0.574
22	CT_218	1.908	0.586	0.408	CT_618	3.288	0.960	0.484
23	CT_147	1.901	0.481	0.365	CT_105	3.192	0.749	0.429
24	CT_229	1.895	0.622	0.391	CT_467	3.184	1.098	0.697
25	CT_639	1.818	0.451	0.330	CT_858	3.107	0.972	0.563
26	CT_249	1.807	0.640	0.369	CT_116	3.088	0.858	0.434
27	CT_001	1.806	0.509	0.322	CT_825	3.073	0.777	0.564
28	CT_115	1.776	0.567	0.353	CT_381	3.044	0.862	0.430
29	CT_346	1.695	0.424	0.284	CT_751	2.912	0.674	0.397
30	CT_579	1.659	0.586	0.317	CT_333	2.865	0.668	0.414
31	CT_584	1.656	0.572	0.379	CT_759	2.854	0.927	0.560
32	CT_795	1.652	0.593	0.276	CT_720	2.824	0.787	0.403
33	CT_307	1.631	0.537	0.284	CT_019	2.794	0.575	0.501
34	CT_703	1.596	0.519	0.292	CT_372	2.774	0.660	0.377
35	CT_541	1.579	0.475	0.314	CT_681	2.723	0.970	0.357
36	CT_181	1.570	0.581	0.333	CT_708	2.695	0.700	0.391
37	CT_228	1.562	0.320	0.276	CT_639	2.675	0.725	0.597
38	CT_381	1.557	0.592	0.314	CT_866	2.665	0.711	0.364
39	CT_814	1.556	0.588	0.286	CT_458	2.635	0.685	0.354
40	CT_048	1.543	0.419	0.301	CT_796	2.634	0.884	0.384

41	CT_313	1.539	0.453	0.282	CT_746	2.609	0.778	0.430
42	CT_741	1.523	0.611	0.297	CT_529	2.587	1.019	0.572
43	CT_372	1.523	0.409	0.260	CT_205	2.535	0.841	0.365
44	CT_414	1.518	0.626	0.281	CT_442	2.517	0.937	0.491
45	CT_388	1.517	0.554	0.313	CT_579	2.517	0.790	0.416
46	CT_681	1.515	0.585	0.276	CT_587	2.442	0.604	0.383
47	CT_232	1.495	0.420	0.291	CT_664	2.435	0.687	0.368
48	CT_567	1.490	0.578	0.292	CT_418	2.400	0.793	0.304
49	CT_802	1.485	0.510	0.277	CT_857	2.374	0.836	0.339
50	CT_526	1.482	0.428	0.273	CT_231	2.314	0.761	0.357
51	CT_691	1.480	0.411	0.282	CT_040	2.302	0.753	0.350
52	CT_110	1.475	0.465	0.251	CT_249	2.253	0.860	0.434
53	CT_659	1.473	0.443	0.273	CT_868	2.250	0.965	0.464
54	CT_853	1.466	0.524	0.290	CT_802	2.244	0.666	0.294
55	CT_300	1.465	0.519	0.280	CT_398	2.238	0.762	0.386
56	CT_009	1.460	0.444	0.261	CT_096	2.237	0.571	0.416
57	CT_081	1.453	0.579	0.270	CT_762	2.226	0.714	0.339
58	CT_353	1.447	0.526	0.286	CT_218	2.211	0.934	0.415
59	CT_601	1.438	0.476	0.325	CT_682	2.203	0.769	0.364
60	CT_830	1.418	0.500	0.265	CT_118	2.180	0.944	0.417
61	CT_844	1.413	0.454	0.331	CT_532	2.167	0.658	0.320
62	CT_482	1.408	0.570	0.299	CT_111	2.164	0.618	0.295
63	CT_330	1.408	0.465	0.309	CT_741	2.136	0.823	0.270
64	CT_186	1.403	0.396	0.264	CT_346	2.135	0.661	0.282
65	CT_067	1.399	0.425	0.229	CT_872	2.113	0.694	0.310
66	CT_398	1.385	0.511	0.285	CT_822	2.030	0.701	0.324
67	CT_159	1.380	0.495	0.266	CT_603	2.016	0.663	0.373
68	CT_597	1.376	0.530	0.259	CT_229	1.955	0.826	0.463
69	CT_458	1.370	0.537	0.269	CT_601	1.954	0.641	0.369
70	CT_751	1.369	0.411	0.269	CT_190	1.951	0.748	0.309
71	CT_602	1.363	0.488	0.272	CT_701	1.940	0.770	0.299
72	CT_569	1.361	0.544	0.259	CT_313	1.936	0.712	0.253
73	CT_503	1.361	0.416	0.258	CT_110	1.894	0.689	0.257
74	CT_325	1.360	0.411	0.273	CT_841	1.821	0.796	0.270
75	CT_756	1.359	0.384	0.268	CT_226	1.802	0.808	0.231
76	CT_789	1.358	0.598	0.274	CT_224	1.799	0.686	0.220
77	CT_657	1.355	0.394	0.276	CT_706	1.710	0.619	0.260
78	CT_143	1.352	0.553	0.313	CT_260	1.675	0.643	0.245
79	CT_587	1.333	0.397	0.273	CT_545	1.662	0.694	0.274
80	CT_538	1.321	0.418	0.245	CT_541	1.661	0.614	0.358
81	CT_129	1.320	0.474	0.261	CT_842	1.640	0.746	0.243
82	CT_118	1.318	0.629	0.320	CT_143	1.636	0.814	0.303
83	CT_603	1.314	0.480	0.281	CT_228	1.629	0.441	0.286
84	CT_480	1.307	0.432	0.242	CT_799	1.601	0.670	0.227
85	CT_421	1.306	0.502	0.258	CT_795	1.601	0.800	0.283
86	CT_772	1.300	0.386	0.280	CT_480	1.586	0.595	0.254

87	CT_444	1.299	0.403	0.267	CT_331	1.571	0.708	0.219
88	CT_173	1.286	0.537	0.253	CT_547	1.570	0.746	0.280
89	CT_724	1.279	0.540	0.255	CT_273	1.534	0.506	0.250
90	CT_829	1.273	0.436	0.245	CT_827	1.520	0.646	0.256
91	CT_532	1.271	0.517	0.308	CT_117	1.506	0.604	0.308
92	CT_449	1.269	0.558	0.249	CT_382	1.478	0.724	0.230
93	CT_568	1.265	0.435	0.274	CT_703	1.459	0.765	0.230
94	CT_433	1.265	0.431	0.265	CT_724	1.458	0.769	0.177
95	CT_342	1.261	0.505	0.227	CT_446	1.438	0.718	0.199
96	CT_231	1.258	0.488	0.297	CT_821	1.429	0.381	0.254
97	CT_457	1.257	0.477	0.219	CT_089	1.421	0.396	0.260
98	CT_529	1.257	0.574	0.329	CT_004	1.418	0.660	0.213
99	CT_446	1.256	0.554	0.277	CT_307	1.404	0.797	0.217
100	CT_832	1.254	0.427	0.275	CT_609	1.388	0.705	0.198
101	CT_080	1.250	0.432	0.272	CT_292	1.371	0.588	0.203
102	CT_841	1.244	0.530	0.275	CT_544	1.367	0.601	0.221
103	CT_357	1.243	0.398	0.261	CT_022	1.358	0.658	0.154
104	CT_281	1.240	0.470	0.268	CT_812	1.352	0.591	0.304
105	CT_847	1.235	0.459	0.258	CT_115	1.336	0.712	0.239
106	CT_539	1.234	0.420	0.230	CT_482	1.322	0.731	0.188
107	CT_528	1.234	0.519	0.260	CT_325	1.317	0.528	0.247
108	CT_797	1.234	0.423	0.243	CT_277	1.315	0.517	0.259
109	CT_076	1.234	0.480	0.287	CT_341	1.308	0.642	0.173
110	CT_440	1.232	0.480	0.259	CT_452	1.302	0.559	0.264
111	CT_737	1.231	0.384	0.251	CT_871	1.302	0.603	0.179
112	CT_481	1.228	0.484	0.244	CT_342	1.301	0.685	0.173
113	CT_277	1.220	0.384	0.270	CT_678	1.284	0.664	0.166
114	CT_212	1.216	0.481	0.227	CT_162	1.283	0.724	0.188
115	CT_192	1.213	0.390	0.225	CT_121	1.280	0.632	0.225
116	CT_612	1.211	0.460	0.239	CT_521	1.273	0.672	0.178
117	CT_873	1.210	0.465	0.310	CT_388	1.268	0.686	0.213
118	CT_563	1.209	0.542	0.269	CT_468	1.268	0.713	0.167
119	CT_272	1.209	0.449	0.239	CT_172	1.256	0.699	0.168
120	CT_172	1.208	0.512	0.237	CT_474	1.253	0.613	0.218

Max= maximum MFI value, mean = mean MFI value, SD = standard deviation
